# Boreal earliest Triassic biotas elucidate globally depauperate hard substrate communities after the end-Permian mass extinction

**DOI:** 10.1038/srep36345

**Published:** 2016-11-08

**Authors:** Michał Zatoń, Grzegorz Niedźwiedzki, Henning Blom, Benjamin P. Kear

**Affiliations:** 1 University of Silesia, Faculty of Earth Sciences, Będzińska 60, 41-200 Sosnowiec, Poland - Centre for Polar Studies KNOW (Leading National Research Centre); 2 Uppsala University, Department of Organismal Biology, Norbyvägen 18A, 752 36 Uppsala, Sweden; 3 Uppsala University, Museum of Evolution, Norbyvägen 16, 752 36 Uppsala, Sweden

## Abstract

The end-Permian mass extinction constituted the most devastating biotic crisis of the Phanerozoic. Its aftermath was characterized by harsh marine conditions incorporating volcanically induced oceanic warming, widespread anoxia and acidification. Bio-productivity accordingly experienced marked fluctuations. In particular, low palaeolatitude hard substrate communities from shallow seas fringing Western Pangaea and the Tethyan Realm were extremely impoverished, being dominated by monogeneric colonies of filter-feeding microconchid tubeworms. Here we present the first equivalent field data for Boreal hard substrate assemblages from the earliest Triassic (Induan) of East Greenland. This region bordered a discrete bio-realm situated at mid-high palaeolatitude (>30°N). Nevertheless, hard substrate biotas were compositionally identical to those from elsewhere, with microconchids encrusting *Claraia* bivalves and algal buildups on the sea floor. Biostratigraphical correlation further shows that Boreal microconchids underwent progressive tube modification and unique taxic diversification concordant with changing habitats over time. We interpret this as a post-extinction recovery and adaptive radiation sequence that mirrored coeval subequatorial faunas, and thus confirms hard substrate ecosystem depletion as a hallmark of the earliest Triassic interval globally.

The end-Permian (P-T) mass extinction 252 Ma was the most severe biotic crisis of the Phanerozoic, initiating wholesale biodiversity collapse^1^,^2^,^3^,^4^,^5^,^6^,^7^. Up to 90% of all marine species are estimated to have disappeared^8^,^9^,^10^,^11^, with synchronous niche loss affecting terrestrial assemblages^12^,^13^,^14^. Although the underlying causes of the P-T event are not completely understood^15^, a primary driver might have been massive volcanic activity within the Siberian igneous province^7^,^16^,^17^,^18^,^19^,^20^. This generated excessive emissions of thermogenic methane, CO_2_ and SO_2_ that cascaded oceanic warming, acidification and anoxia/euxinia with accompanying bio-productivity decline^10^,^16^,^21^,^22^,^23^,^24^,^25^,^26^,^27^,^28^.

Ecosystem recovery after the P-T interval was seemingly protracted, but this has been reconstructed largely from the well-documented record of soft-bottom marine organisms^29^,^30^,^31^,^32^. In contrast, corresponding hard substrate assemblages remain enigmatic, primarily because of limited sampling. To date, the most informative fossils derive from the subequatorial shallow Panthalassic basins bordering Western Pangaea^33^, and the Tethyan margin of the South China Craton^34^,^35^. These collectively infer disappearance of rich latest Permian encrusting benthos, and its subsequent replacement in the earliest Triassic by monogeneric colonies of microconchid tubeworms – an extinct suspension-feeding clade possibly related to ‘lophophorates’^36^,^37^. However, the broader palaeobiogeographical continuity of these successions is speculative, especially with regard to mid-high palaeolatitude (>30°N) faunas from the Boreal Realm. These occupied a completely separate bio-region^38^ and are thus crucial for establishing globally continuous patterns. Here we document the first Early Triassic Boreal hard substrate benthic assemblages from East Greenland, a remote landmass that preserves one of the most extensive P-T marine rock sections known worldwide^12^,^39^. Our new fossils reveal compatible microconchid predominance, but with a unique Boreal signature of morphological diversification across changing depositional settings; this not only elucidates distinctive regional endemism, but also opportunistic ecosystem expansion during the initial recovery phase after the P-T boundary.

## Results

### Lithostratigraphy and palaeoenvironment

**Figure 1:**
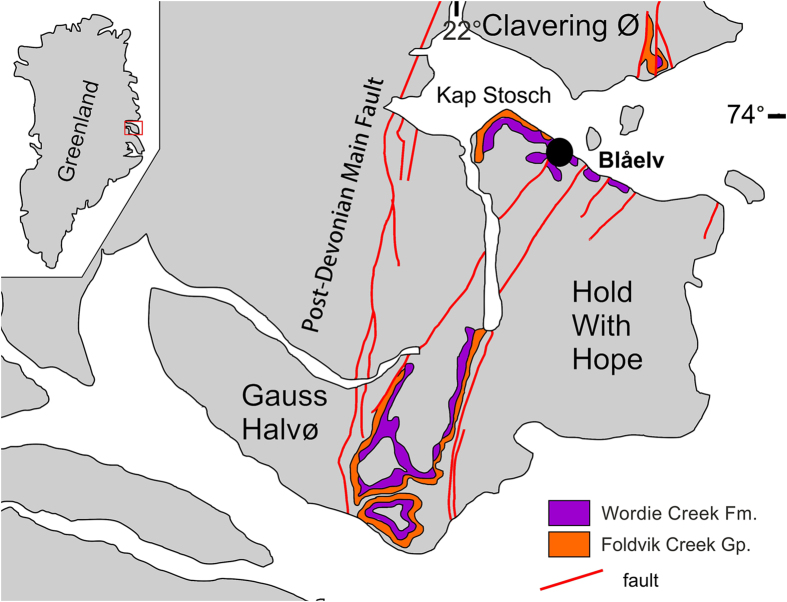
Locality. Map of Greenland with enlargement of the Hold with Hope peninsula showing with the Kap Stosch field site (black circle). Modified from Nielsen^42^ and Bjerager *et al*.^39^. Graphics created by G.N. in *CorelDraw* 11, www.coreldraw.com.

**Figure 2:**
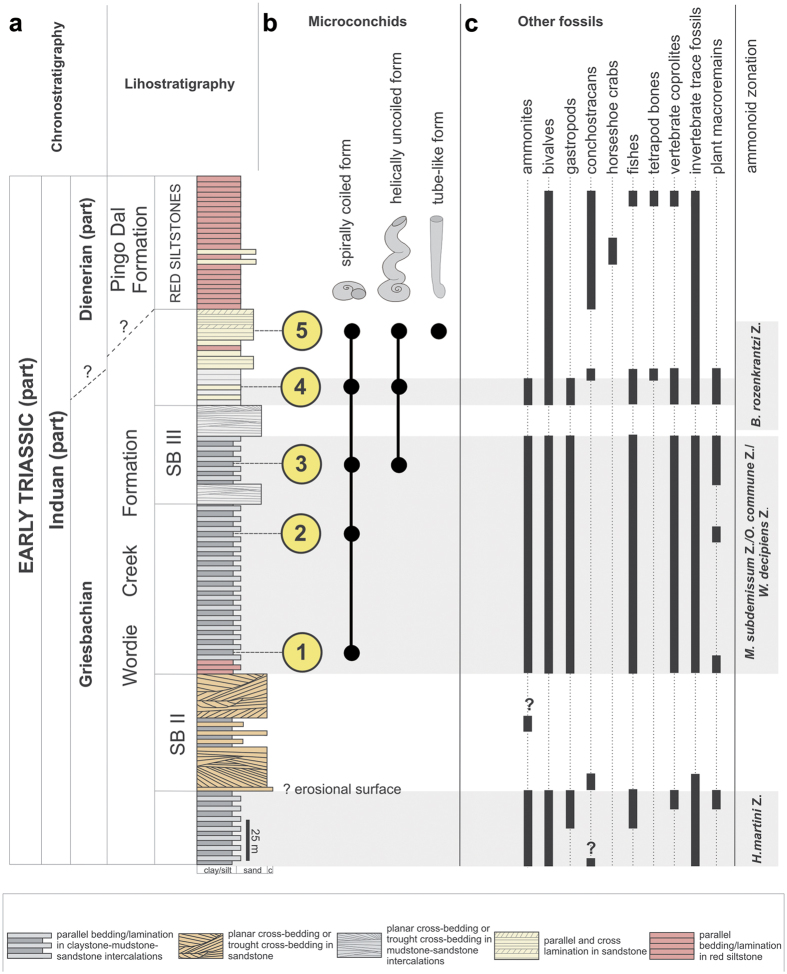
Lithostratigraphy and fossil content. (**a**) Schematic section of the lowermost Triassic succession at Kap Stosch with (**b**) microconchid morphotypes recovered from intervals 1–5. (**c**) Stratigraphic occurrence of associated fossils. SB II and SB III indicate major sandstone facies. Compiled and graphically drawn by G.N. in *CorelDraw* 11, www.coreldraw.com.

**Figure 3:**
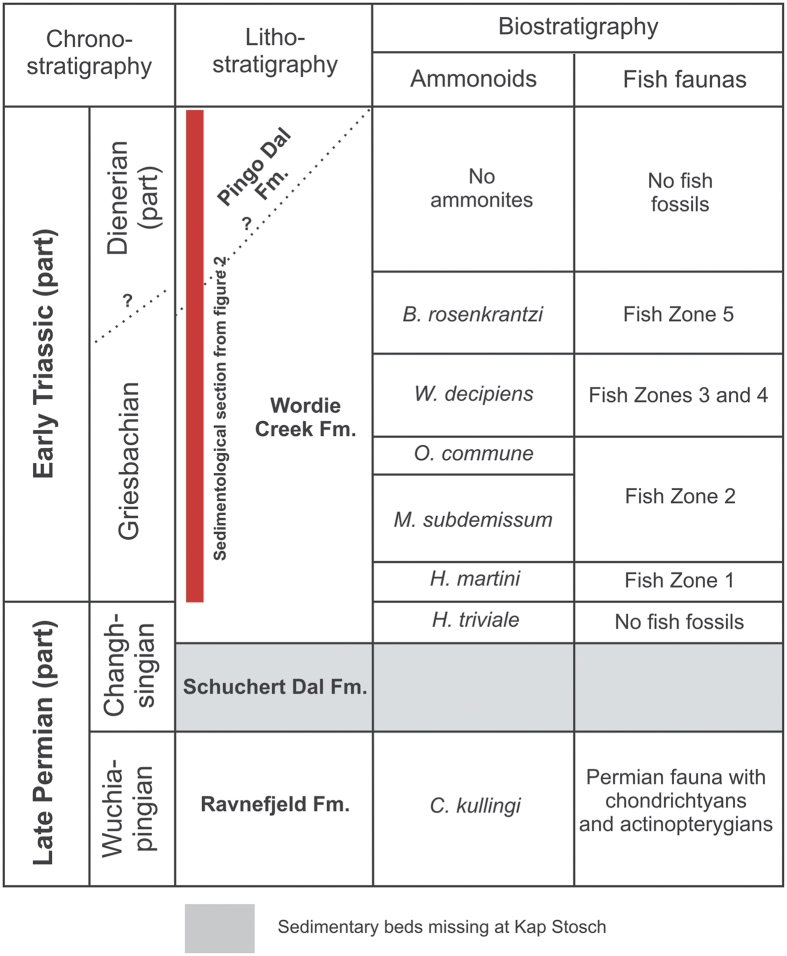
Biostratigraphical correlation. Upper Permian–Lower Triassic ammonoid and fish zonation^39^,^42^,^43^ from Kap Stosch, East Greenland. Not depicted to scale. Compiled and graphically drawn by G.N. in *CorelDraw* 11, www.coreldraw.com.

We systematically collected 131 bivalve shell samples and five large rock slabs, all with encrusted microconchids, from a ∼600 m transect through the Wordie Creek Formation^39^,^40^,^41^. This was exposed along the Blue River (Blåelv) and adjacent Stensiö Plateau at Kap Stosch on the Hold with Hope peninsula of East Greenland^42^ (Fig. 1). During the latest Permian–Early Triassic, this landmass formed part of the northwestern coastal margin of Pangaea verging on the Boreal Sea. Thick siliciclastic sequences accumulated as sub-basin infills within the burgeoning rift zone between Greenland and Norway^41^,^43^,^44^,^45^. Evidence of these deposits is today preserved at Kap Stosch (Fig. 2), as well as laterally equivalent localities on Geographical Society Ø, Traill Ø, Wegener Halvø and Jameson Land^39^. The lowermost beds in the Blue River section comprise the Upper Permian (Wuchiapingian) Ravnefjeld Formation, which is unconformably overlain by a shore-face*–*prograding deltaic sequence containing ammonoids (*Hypophiceras triviale–Hypophiceras martini* zones: *sensu* Bjerager *et al*.^39^), and constitutes the basal horizon of the Wordie Creek Formation (Fig. 3). This earliest Triassic (Induan) unit successionally trends through marine shales and mudstones with sandy–conglomeratic turbidites in the lower–middle Griesbachian *Metophiceras subdemissum–Ophiceras commune* ammonoid zones, to mudstones and shore-face sandstones with fluvial conglomerate in the mid–upper Griesbachian to Dienerian *Wordieceras decipiens*–*Bukkenites rosenkrantzi* ammonoid zones^39^. Dienerian strata of the *Anodontophora breviforma*–*Anodontophora fassaensis* bivalve zones represent tidally-influenced paralic sandstones and overlying terrestrial red siltstones with poorly developed palaeosols, bivalves, numerous invertebrate traces, conchostracans and aquatic vertebrate remains^42^,^43^. Two thick sandstone bodies (SB II and SB III) also intercalate within these sediments, which are characteristically rich in fish fossils (actinopterygians and coelacanths) that provide a readily identifiable cross-referencing field zonation (Fish zones 1–5: *sensu* Nielsen^42^).

We examined five discrete fossiliferous intervals (herein numbered 1–5: Fig. 2; see also Supplementary Fig. S1) within the Wordie Creek Formation, which yielded microconchids from the *M. subdemissum*, *O. commune*, *W. decipiens* and *B. rosenkrantzi* ammonoid zones, and *A. breviforma* bivalve zone respectively (Figs 2 and 3). Lithologically these horizons comprised mudstones with abundant bivalves, and locally occurring *Archaeolithophyllum* boundstones (=red algal carbonates) grading upwards into fine-grained sandstones. *Archaeolithophyllum* algae have also been reported in the earliest Triassic deposits of Jameson Land further to the south.^46^. Dimensionally very small pyrite framboids were recovered from intervals (1) – (*M* = 6.8 μm; *SD* = 1.5), (3) – (*M* = 4.4 μm; *SD* = 1.8), and (4) – (*M* = 5.6 μm; *SD* = 1.4). These infer dysoxic (1), to anoxic (3–4) bottom waters^23^, and concur with prolific occurrences of the dysaerobic soft-substrate bivalve *Claraia*^46^. The smallest framboid diameters were derived from the *Archaeolithophyllum* boundstones of interval (3), implying microbe-enriched local anoxia. In contrast, the absence of framboids from intervals (2) and (5), coupled with prevalent crystalline pyrite, suggests periodic oxic fluctuations^23^.

### Microconchid assemblages

**Figure 4:**
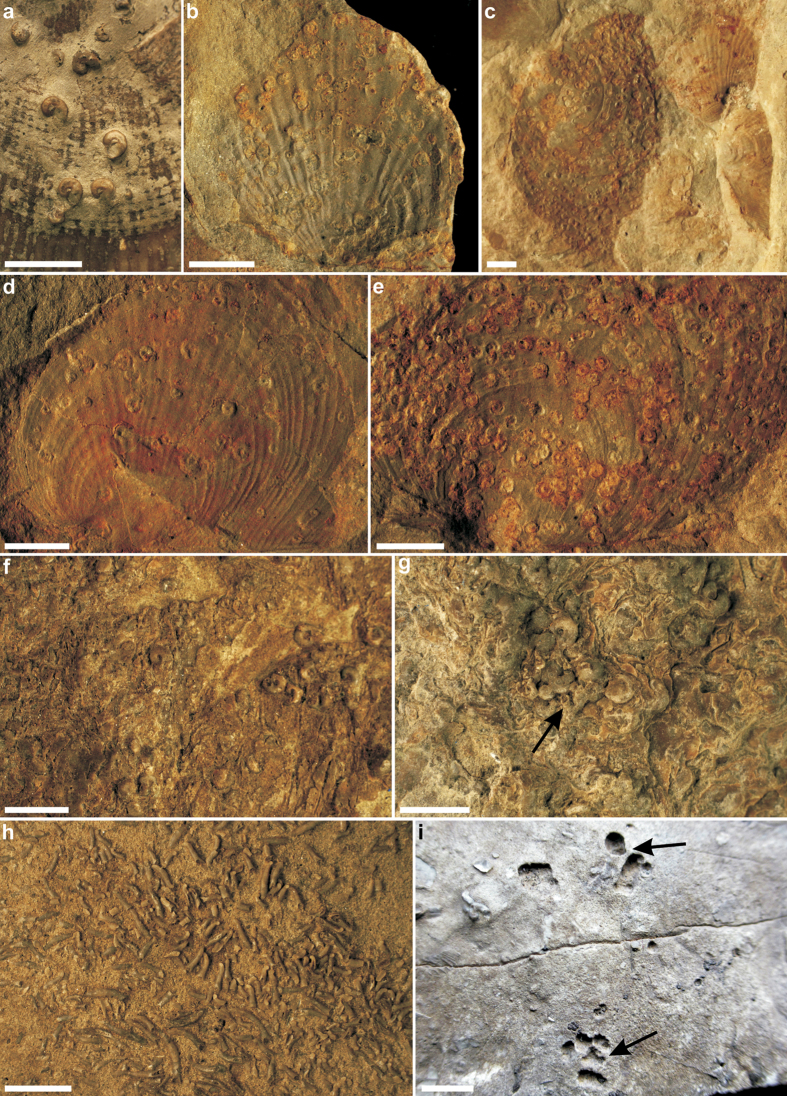
Triassic microconchids from Kap Stosch, East Greenland. (**a**) *Microconchus* encrusting *Claraia* shells from interval (1) (PMU 28936). (**b–e**) *Microconchus* encrusting *Claraia* shells from interval (2): (**b**) PMU 28942; (**c–e**) PMU 28940. (**f,g**) *Microconchus* encrusting *Archaeolithophyllum* boundstone from interval (3) (PMU 28954, PMU 28950); black arrow in (**g**) indicates an uncoiled tube. (**h**) Dense accumulation of *Spathioconchus weedoni* gen. et sp. nov. distributed across mudstone bedding planes from interval (5) (PMU 28962). (**i**) *Entobia*-like borings on a carbonate clast from interval 3 (PMU 28951). Scale bars 5 mm.

**Figure 5:**
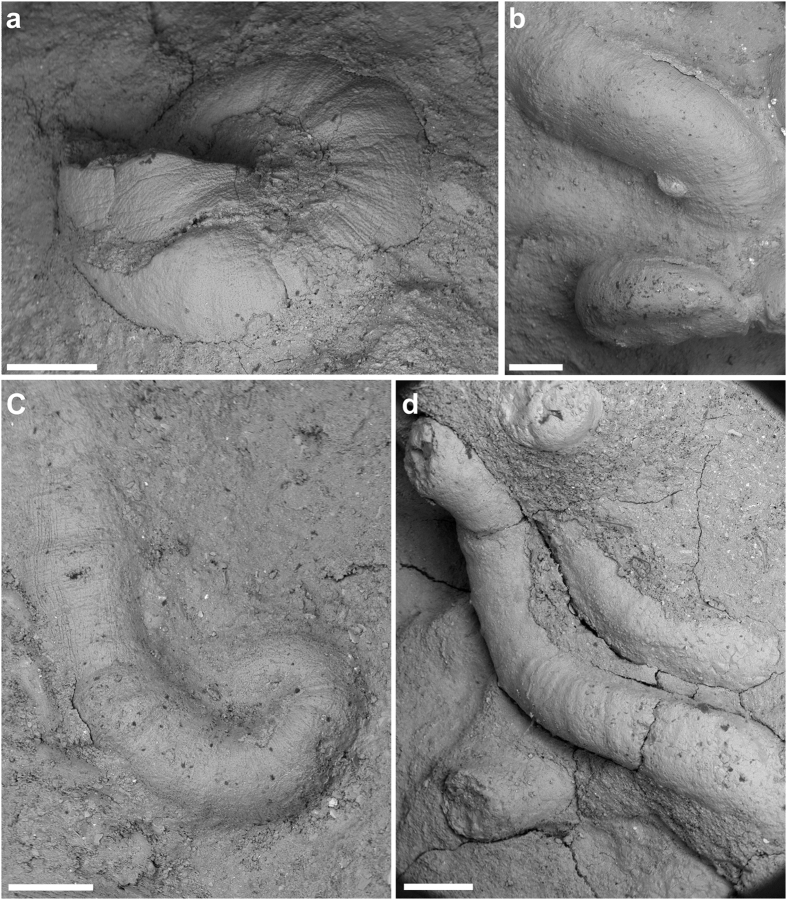
ESEM images of selected microconchids. (**a**) Spirally coiled *Microconchus* encrusting *Claraia* shells from interval (3) (PMU 28949). (**b,c**) Helically uncoiled *Microconchus* from interval (3) (PMU 28951). (**d**) Helically uncoiled *Microconchus* from interval (5) (PMU 28961). Scale bars 500 μm.

Our microconchid fossils were found attached to both *Claraia* shells and boundstones (Fig. 4). No other encrusting organisms were detected except for shallow (~1 mm deep) borings possibly attributable to the clionaid poriferan ichnite *Entobia* (Fig. 4); these were associated with a single clast from interval (3). Microconchids occurring in the boundstones infested thin calcitic sheets of *Archaeolithophyllum* (see thin section in Supplementary Fig. S2). Density of the microconchid encrustations ranged from 8–83 tubes/cm^2^ across all substrates, with the most prolific coverage on *Claraia* shells in intervals (1) *M* = 29.4 tubes/cm^2^, and (2) *M* = 22.9 tubes/cm^2^. Colonies attached to boundstones alternatively averaged only 11.8 tubes/cm^2^, but this could reflect under-sampling of the unexposed bedding planes. Abundant microconchids (*M* = 29.4 tubes/cm^2^) were also scattered randomly across the mudstone layers in interval 5 (Figs 4 and 5; see also Supplementary Fig. S3), but these had likely detached (as implied by their smooth undamaged bases and lack of adhering particles: Fig. 5) from consolidated organic substrates such as algal filaments or microbial mats^47^.

**Figure 6:**
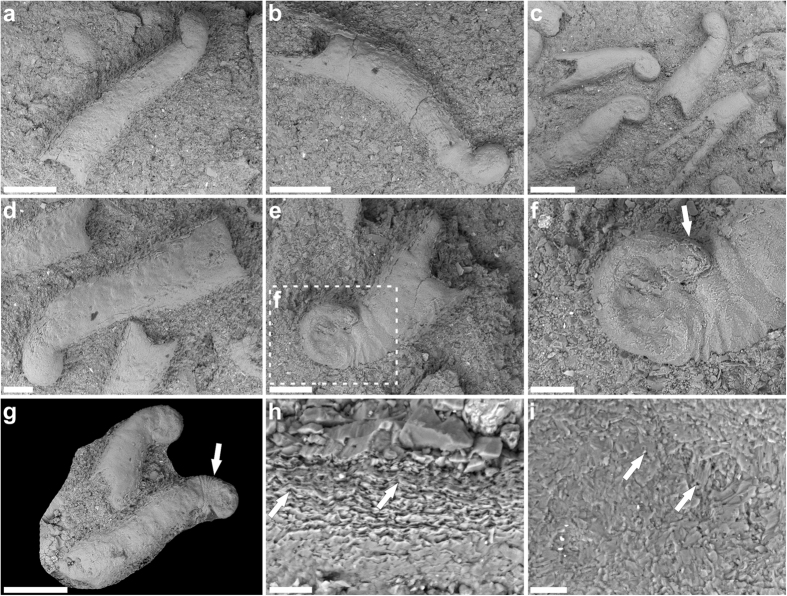
Spathioconchus weedoni gen. et sp. nov. (**a–d**) Examples showing conical tube form with small attachment base, smooth external surface and (**d**) adaperturally concave riblets. (**e,f**) Enlargement of basal attachment showing characteristic ‘nucleus’ (arrowed). (**g**) Holotype PMU 28962a bearing riblets at attachment base/tube border (arrowed). (**h**) Microlamellar tube structure interrupted by prominent punctae (arrowed). (**i**) Diminutive punctae (arrows) on the exfoliated tube exterior. PMU 28962. Scale bars (**a–c**,**g**) 500 μm, (**d,e**) 200 μm, (**f**) 100 μm, (**h,i**) 20 μm.

Individual microconchid tube shapes also varied substantially between colonies, as well as across different substrates and intervals (Fig. 2). For example, those adhering to *Claraia* shells in intervals (1–5) formed squat spiral tubes, whereas those attached to algal filaments within boundstones from interval (3) displayed both spiral and helically uncoiled tubes (Fig. 5). Microconchids dispersed across mudstone layers in interval (5) were helically uncoiled and upright with a convolutional basal attachment (Fig. 5). Their external ornamentation was, nonetheless, identical with fine transverse riblets and lateral striae, accompanied by prominent punctae that penetrated the microlamellar tube structure (Supplementary Fig. S4); these features are taxonomically consistent with the genus *Microconchus* and species such as *M*. *valvatus*^48^. On the other hand, a unique conical tube morphotype with diminutive globular attachment area (Fig. 6) was recovered from fine-grained facies in interval (5). Its distinctive shape, coupled with unusual attachment base, surface ornamentation and tube structure comprising coarse transverse ridges and minute punctae serve to diagnose a new taxon *Spathioconchus weedoni* gen. et sp. nov. (see Supplementary Note). Structural diversity in microconchid tubes^49^,^50^,^51^ has elsewhere been attributed to prevailing environment^52^, with progressive uncoiling being an adaptation to avoid burial in accumulating sediment^49^, or overgrowth by accreting microbial mats^34^. We further interpret the reduced attachment area and peculiar conical form of *S. weedoni* as characteristics of an upright life position and densely packed colonial arrangement.

## Discussion

**Figure 7:**
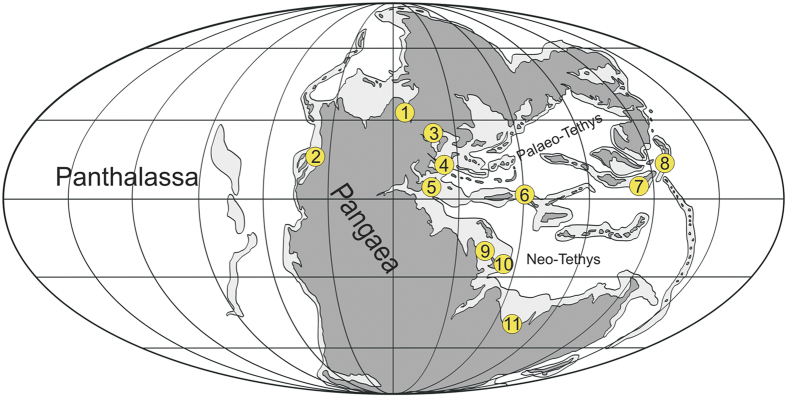
Palaeogeographic distribution of Early Triassic microconchids. 1. East Greenland^12^,^53^,^54^. 2. Western USA^33^,^60^. 3. Poland^47^. 4. Hungary^66^. 5. Italy^8^,^74^,^75^. 6. Iran^74^. 7. South China^34^,^35^,^76^,^77^,^78^. 8. Southwest Japan^79^. 9. Persian Gulf 80. 10. Oman^81^. 11. Australia^82^. Palaeogeographic map from Blakey^83^. Graphics created by G.N. in *CorelDraw* 11, www.coreldraw.com.

The prevalence of microconchids in offshore and lower shore-face settings bordering Western Pangaea provided the original basis for positing global impoverishment of hard substrate marine ecosystems during the earliest Triassic^33^. Indeed, prolific microconchids have since been found in shallow and deep-water microbialites, on *Claraia* shells and bioclastic limestones^34^,^35^ throughout the Tethyan Realm (Fig. 7). These mainly subequatorial palaeogeographical records are complimented here by the first equivalent Boreal occurrences, which demonstrate a coherent signature of microconchid ecosystem dominance and a near total dearth of other skeletonized encrusting organisms. Previous reports of the serpulid polychaetes *Spirorbis* and *Serpula* from Kap Stosch^53^,^54^ and Jameson Land^12^ in East Greenland can be confidently re-identified as microconchids^36^. The only other hard substrate faunal element in our samples was the *Entobia*-like ichnite, which equates to the possible phoronid boring *Talpina* from Western Pangaea^33^; however, these traces are extremely rare suggesting that endoliths, when present, constituted a minute component of the entire biotic assemblage.

In addition to their numerical abundance, both the structural and taxonomic diversity of Boreal microconchids noticeably increased over time: Griesbachian assemblages of coiled *Microconchus* being restricted to *Claraia* shells, but subsequently replaced by sympatric colonies of coiled and helically uncoiled *Microconchus* attached to *Claraia* and algal layers. Finally, conical *Spathioconchus weedoni* appeared as a novel element in mudstone facies from the early Dienerian onwards. This clear eco-morphological trending infers progressive adaptation and habitat expansion, incorporating an innovative dispersal into localized paralic conditions. Interestingly, Yang *et al*.^35^ described similar coeval microconchid morphotypes from Griesbachian microbialites in South China. Their specimens included spiral and helically uncoiled forms resembling *Microconchus utahensis*, *Helicoconchus elongatus*, and *M. aberrans* respectively. Compatible structural diversification thus seems to have occurred in both the Boreal-Panthalassan and Tethyan realms, where microconchids paralleled stromatolites^55^, inarticulate brachiopods^56^, *Claraia* bivalves^4^,^5^,^57^ and various foraminiferans^58^ as opportunistic occupiers of benthic marine ecospace in the earliest Triassic^33^,^34^,^35^. By the Spathian (late Olenekian), however, bivalves and foraminiferans, together with boring suspension-feeders^33^,^59^ had colonised hard substrates to create transient metazoan reefs; these established on multi-taxic sponge^60^ and bivalve^61^ frameworks during the Smithian–Spathian “coral gap”^10^,^11^,^60^. The recovery of lower tier, skeletonised biotas was therefore evidently delayed in comparison to soft-bottom communities^31^,^32^,^62^, a phenomenon that we show manifested simultaneously within both low, and mid-high palaeolatitude assemblages. Certainly, some filter feeding organisms such as crinoids seem to have re-diversified earlier in the Boreal Realm (at least after the latest Induan *A. fassaensis* bivalve zone equivalent^63^), yet typical encrusters including cyclostome bryozoans and serpulid polychaetes did not fully re-establish until the Rhaetian^64^. This conspicuous underrepresentation – which is likely not taphonomic because Palaeozoic encrusters possessed similar calcitic skeletons^65^ and cemented to the substrate throughout the sessile phase of their life cycle – accords with extreme fluctuations in oceanic salinity^66^, de-oxygenation^9^,^40^,^67^, intense weathering and run-off that are thought to have promoted widespread eutrophication and the proliferation of stromatolite-forming microbial substrates^10^,^11^,^26^,^68^ in the absence of mat-grazing organisms^55^,^69^. Moreover, these markedly atypical conditions apparently favoured microconchids, which were ubiquitous across marine to brackish and even freshwater habitats^36^,^70^,^71^ and readily colonized microbial/algal substrates, perhaps because of their stability and immediate supply of nutrients and oxygen^34^,^72^. The propagation of these environments during the earliest Triassic could therefore explain the selective survival of microconchids versus other encrusting benthos, and otherwise reflects the ecosystem homogeneity that characterised the post P-T interval on a global scale.

## Methods

Our microconchid fossils were inspected using both a binocular microscope and Philips XL30 Environmental Scanning Electron Microscope (ESEM) installed at the Faculty of Earth Sciences, University of Silesia, Sosnowiec, Poland. Uncoated ESEM samples were examined with back-scattered (BSE) imaging under low vacuum conditions. Samples for pyrite framboids were thin-sectioned and assessed by ESEM. More than 50 specimens were measured and interpreted following the procedures of Wignall and Newton^73^ and Bond and Wignall^23^. All fossils documented herein are housed in the Palaeontology Collection at Museum of Evolution, Uppsala University (PMU), Sweden under the registration numbers PMU 28933–PMU 28963.

## Additional Information

**How to cite this article**: Zatoń, M. *et al*. Boreal earliest Triassic biotas elucidate globally depauperate hard substrate communities after the end-Permian mass extinction. *Sci. Rep*. **6**, 36345; doi: 10.1038/srep36345 (2016).

**Publisher’s note:** Springer Nature remains neutral with regard to jurisdictional claims in published maps and institutional affiliations.

## Supplementary Material

Supplementary Information
