# Supplementary material for: Boreal earliest Triassic biotas elucidate globally depauperate hard substrate communities after the end-Permian mass extinction
Source: Sci Rep. 2016 Nov 8;6:36345. doi: 10.1038/srep36345 (PMC5099577; doi:10.1038/srep36345)
Supplement: Supplementary Information [file srep36345-s1.doc]

Supplementary Information

**Boreal earliest Triassic biotas elucidate globally depauperate hard substrate communities after the end-Permian mass extinction**

Michał Zatoń^1^, Grzegorz Niedźwiedzki^2^, Henning Blom^2^, Benjamin P. Kear^3^

^1^University of Silesia, Faculty of Earth Sciences, Będzińska 60, 41-200 Sosnowiec, Poland - Centre for Polar Studies KNOW (Leading National Research Centre)

^2^Uppsala University, Evolutionary Biology Center, Department of Organismal Biology, Norbyvägen 18A, 752 36 Uppsala, Sweden

^3^Uppsala University, Museum of Evolution, Norbyvägen 16, 752 36 Uppsala, Sweden.

*Corresponding author: e-mail: mzaton@wnoz.us.edu.pl

**Supplementary Note**

Class Tentaculita Bouček, 1964^1^

Order Microconchida Weedon, 1991^2^

Genus ***Spathioconchus*** gen. nov.

*Etymology*: In honour of Leonard F. Spath (1882-1957), who undertook the seminal investigations on the Early Triassic fossils of East Greenland, and *conch* – a tubular shell (masculine gender).

*Type species*: *Spathioconchus weedoni* sp. nov.

*Diagnosis*: Straight to slightly curved conical tubes with extremely small globular attachment base and minute punctae penetrating the tube wall.

***Spathioconchus weedoni*** sp. nov.

*Etymology*: In honour of Michael J. Weedon, who established the Order Microconchida.

*Holotype*: PMU 28962a (Fig. 5g).

*Referred material*: Additional individuals PMU 28962b–PMU 28962i as depicted in Fig. 5a–f.

*Diagnosis*: As for genus.

*Description*: Elongate conical tube (progressively widening with growth); straight to slightly curved with an oval cross section. External ornamentation consists of coarse transverse ridges that are slightly concave adaperturally. Tube base (‘protoconch’) is small (ca. 250 µm in diameter) and globular with a smooth exterior. Attachment surface bears a noticeably elongated ‘nucleus’. A few thin, densely spaced ridged mark the surface area above the globular base. The interior tube space is devoid of septa. The tube microstructure is lamellar and interrupted by distinct punctae that emerge as micron-sized pores on the exfoliated exterior tube surface.

*Remarks*: The conspicuous conical tube form with small basal attachment and distinct ‘nucleus’ clearly differentiates *Spathioconchus weedoni* from microconchid taxa manifesting similar uncoiled tube structures – e.g., some *Palaeoconchus*^3^ and *Microconchus*^4-5^; especially the sympatric *Microconchus* from Kap Stosch interval 5, which are more robust and have larger punctae (Supplementary Fig. S4c-d). The lack of internal septa, as well as lateral budding and binary fission between tubes further distinguishes *S. weedoni* from the helix-shaped *Helicoconchus elongatus* from the Lower Permian of Texas^6^.

*Stratigraphical and geographical range*: *Anodontophora breviforma* bivalve zone, mid-upper Induan (Dienerian), lowermost Triassic at Stensiö Plateau, Kap Stosch, Hold With Hope peninsula, East Greenland.

**Supplementary Figures**

**
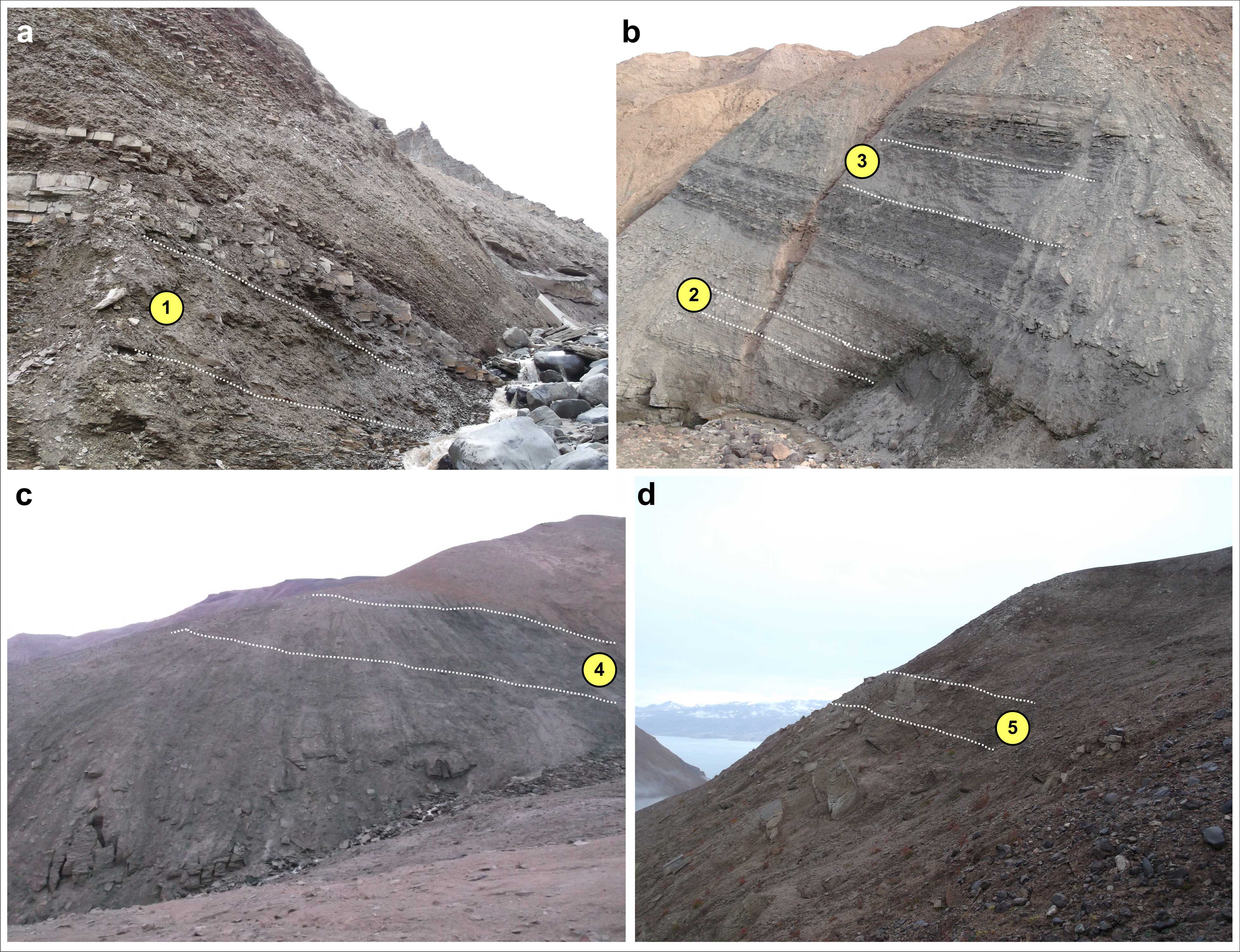
**

**Supplementary Figure S1. Field photographs taken at Kap Stosch, East Greenland showing fossiliferous intervals 1–5.** (a) Lower Blue River section with interval (1). (b) Middle Blue River section with interval (2) and (3); these are otherwise rich in *Claraia* and small gastropods. (c) Upper Blue River section with interval (4) exposed on the western slope of the Stensiö Plateau. (d) Uppermost interval (5) with red beds on the western slope of the Stensiö Plateau. Photographs by Grzegorz Niedźwiedzki.


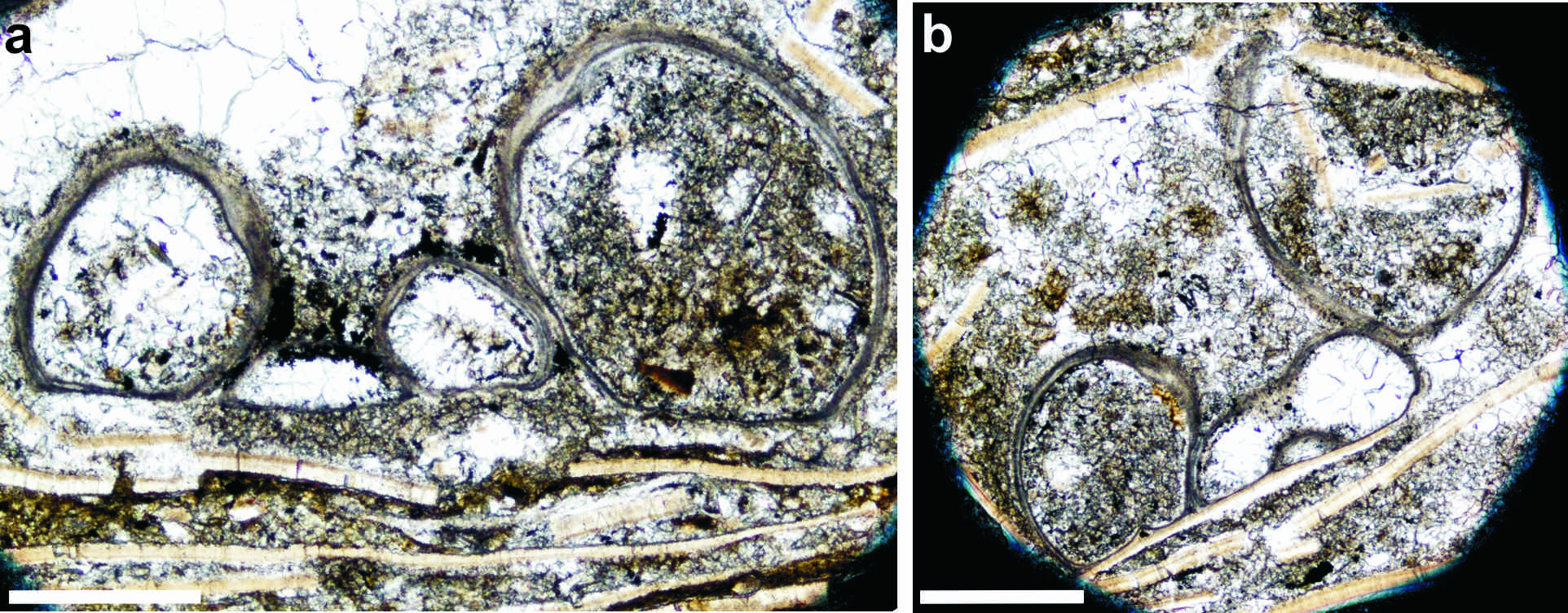


**Supplementary Figure S2. Thin section of *Archaeolithophyllum* boundstone showing microconchids attached to algal filaments.** Scale bar 500 μm.


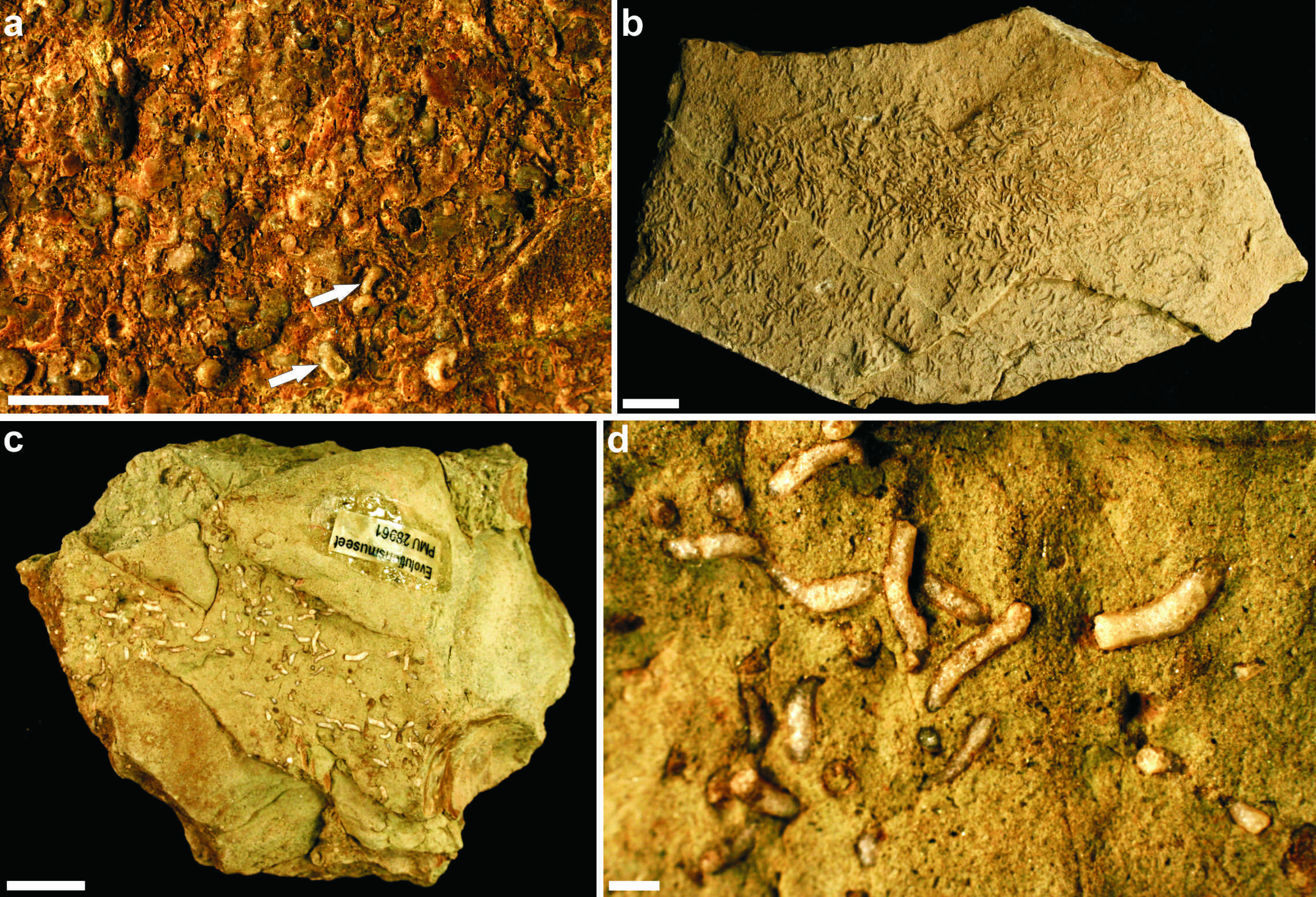


**Supplementary Figure S3. Microconchids preserved on various substrates.** (a) *Archaeolithophyllum* boundstone from interval (3) with uncoiled *Microconchus* tubes (arrows). (b) Dense accumulation of *Spathioconchus weedoni* sp. nov. tubes preserved on mudstone slabs from the interval (5). (c–d) Helically uncoiled *Microconchus* tubes on mudstone from interval (5). Scale bars (a) 5mm, (b–c) 10 mm, (d) 1 mm.

**
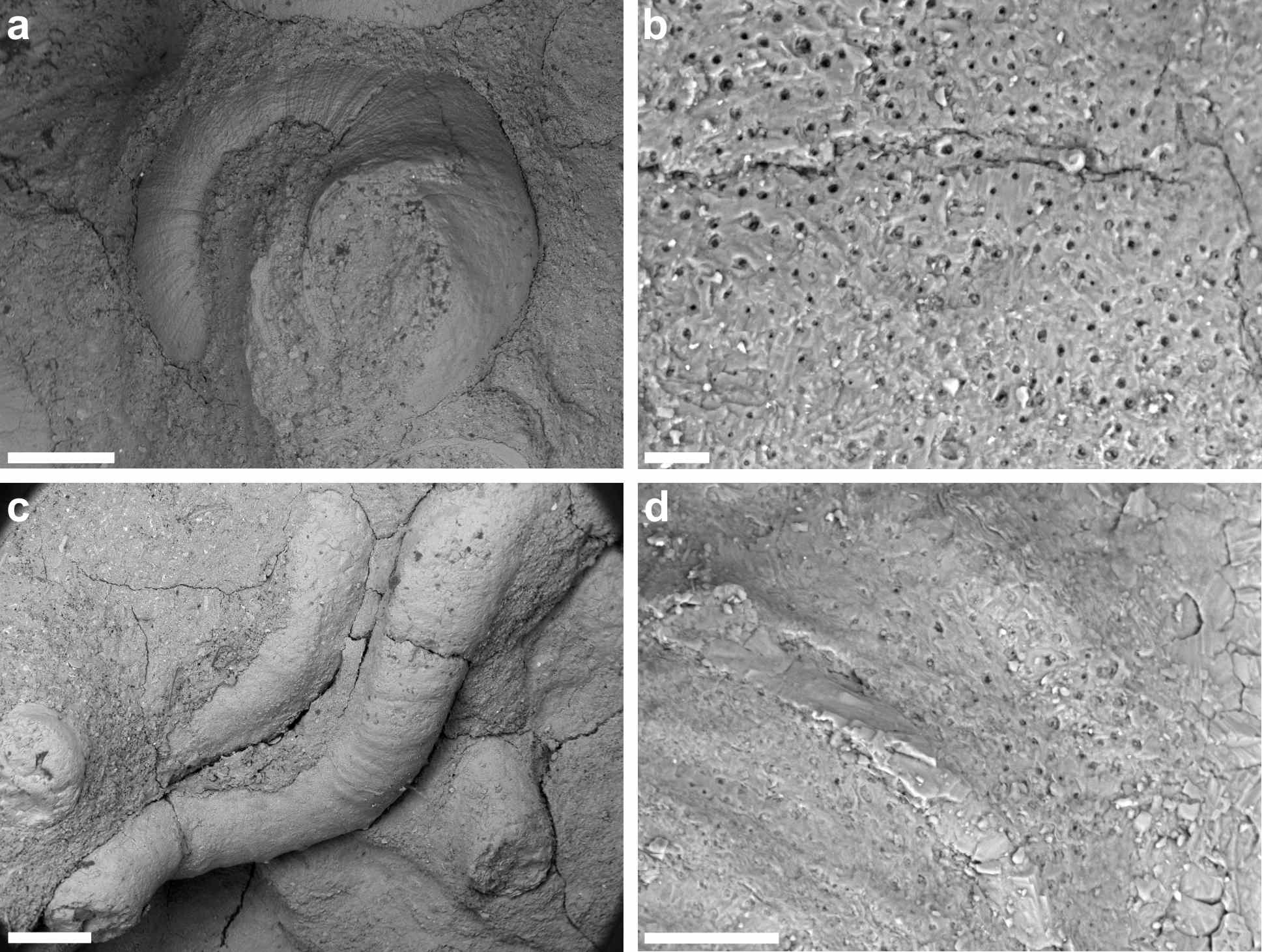
**

**Supplementary Figure S4. Microconchid tube punctation.** (a) Spirally coiled *Microconchus* from interval (3) with (b) enlargement of punctations. (c) Helically uncoiled *Microconchus* from interval (3) with (d) enlargement of punctations. Note compatible morphologies in both specimens Scale bars, (a, c) 500 μm, (b) 20 μm, (d) 50 μm.

**References**

1. Bouček, B. *The* Tentaculites *of Bohemia* (Czechoslovakian Academy of Sciences, Prague, 1964).
2. Weedon, M. J. Microstructure and affinity of the enigmatic Devonian tubular fossils *Trypanopora*. *Lethaia* **24**, 223–227 (1991).
3. Zatoń, M. & Krawczyński, W. New Devonian microconchids (Tentaculita) from the Holy Cross Mountains, Poland. *J. Paleontol*. **85**, 757–769 (2011).
4. Zatoń, M. & Peck, R. L. Morphology and palaeoecology of new, non-marine microconchid tubeworm from Lower Carboniferous (Upper Mississippian) of West Virginia, USA. *Ann. Soc. Geol. Polon*. **83**, 37–50 (2013).
5. Vinn, O. Shell structure of helically coiled microconchids from the Middle Triassic (Anisian) of Germany. *Paläont. Zeit*. **84**, 495–499 (2010).
6. Wilson, M. A., Vinn, O. & Yancey, T. E. A new microconchid tubeworm from the Artinskian (Lower Permian) of central Texas, USA. *Acta Palaeontol. Pol.* **56**, 785–791 (2011).
